# Effects of a 12-Week Suspension versus Traditional Resistance Training Program on Body Composition, Bioimpedance Vector Patterns, and Handgrip Strength in Older Men: A Randomized Controlled Trial

**DOI:** 10.3390/nu13072267

**Published:** 2021-06-30

**Authors:** Francesco Campa, Brad J. Schoenfeld, Elisabetta Marini, Silvia Stagi, Mario Mauro, Stefania Toselli

**Affiliations:** 1 Department of Life Quality Studies, University of Bologna, 47921 Rimini, Italy; 2 Department of Health Sciences, CUNY Lehman College, Bronx, NY 10468, USA; bradschoenfeldphd@gmail.com; 3 Department of Life and Environmental Sciences, University of Cagliari, Cittadella Universitaria, Monserrato, 09042 Cagliari, Italy; emarini@unica.it (E.M.); silviastagi@unica.it (S.S.); 4 Department of Basic Medical Sciences, Neurosciences and Sense Organs, University of Study of Bari, 70121 Bari, Italy; mario.mauro.194@gmail.com; 5 Department of Biomedical and Neuromotor Sciences, University of Bologna, 40126 Bologna, Italy; stefania.toselli@unibo.it

**Keywords:** bioelectric impedance analysis, BIVA, muscle mass, fat mass, phase angle

## Abstract

This investigation aimed to compare the effects of suspension training versus traditional resistance exercise using a combination of bands and bodyweight on body composition, bioimpedance vector patterns, and handgrip strength in older men. Thirty-six older men (age 67.4 ± 5.1 years, BMI 27.1 ± 3.3 kg/m^2^) were randomly allocated into suspension training (*n* = 12), traditional training (*n* = 13), or non-exercise (*n* = 11) groups over a 12-week study period. Body composition was assessed using conventional bioelectrical impedance analysis and classic and specific bioelectric impedance vector analysis, and handgrip strength was measured with a dynamometer. Results showed a significant (*p* < 0.05) group by time interaction for fat mass, fat-free mass, total body water, skeletal muscle index, classic and specific bioelectrical resistance, classic bioelectrical reactance, phase angle, and dominant handgrip strength. Classic and specific vector displacements from baseline to post 12 weeks for the three groups were observed. Handgrip strength increased in the suspension training group (*p* < 0.01, ES: 1.50), remained stable in the traditional training group, and decreased in the control group (*p* < 0.01, ES: −0.86). Although bodyweight and elastic band training helps to prevent a decline in muscle mass and handgrip strength, suspension training proved more effective in counteracting the effects of aging in older men under the specific conditions studied.

## 1. Introduction

It is predicted that the age distribution of the world’s population will increase from 727 million people aged over 65 years in 2020 to 1.5 billion people by 2050 [1]. Aging is accompanied by a decline in body composition and strength [2], which increases the risk of age-related diseases [3]. An excess of fat mass correlates with a higher cardiometabolic risk, while reductions in body fluids can compromise hydration and nutritional status [4,5]. The age-related loss of muscle quantity and strength results in a condition termed sarcopenia, which is associated with a variety of detrimental health outcomes such as disability, morbidity, mortality, and a lower quality of life [3]. As a result, there is difficulty in neuromuscular activity and contractility of muscle fibers and impaired muscle strength [6]. In this sense, muscle quantity and strength are proposed by European Working Group on Sarcopenia in Older People (EWGSOP) 2019 as important factors to be considered to identify sarcopenia [3]. Additionally, muscle strength is recognized as a better predictor of adverse health outcomes than muscle mass [3]. In this regard, handgrip strength evaluation is commonly used in health care administration and is included among the tests for diagnosing sarcopenia [3]. As results, a subject can gain an advantage by changing their body composition features and diet habits [4,5].

Monitoring body composition has become crucial, and assessing it appropriately allows for an accurate evaluation of nutritional status in elderly people. In this context, the bioelectrical impedance analysis (BIA) is one of the most popular methods used to assess body composition, primarily because of its combination of cost-efficiency, user friendliness, and portability [7,8,9]. By employing bioimpedance-based predictive equations, it is possible to estimate and monitor changes in body composition parameters such as fat mass, total body water, and muscle mass [10]. In addition to the estimation of body composition, it is possible to evaluate raw bioelectrical parameters such as resistance (R), reactance (Xc), and phase angle. The evaluation of R and Xc can be performed through bioelectrical vector analysis (BIVA), in which the two parameters are represented as a vector within a graph [11]. According to this method, if R and Xc are standardized for height (classic BIVA), they are informative of changes in total body water, through changes in the length of the vector [11]. Alternatively, by adjusting R and Xc for body geometries (specific BIVA), the length of the vector becomes informative for the percentage of fat mass but not total body water, while the lateral displacements of the vector, similar to the classical BIVA, reflect the changes in phase angle [12]. Furthermore, BIVA provides the ability to plot the vector on tolerance ellipses representing the bivariate distribution for R and Xc of the reference population [12,13].

Inactivity seems to be a factor that accelerates the decline in body composition and decrease in body function, while regular physical exercise can slow down these processes. Although physical training has been suggested to promote physical and mental health [14] during the COVID-19 outbreak, especially in older adults [15], measures to contain the spread of the virus have mainly involved social distancing, and the closure of leisure and sports facilities. Therefore, developing an alternative home-based training strategy is urgently needed to maintain regular physical activity during the time of pandemic [16,17]. Accordingly, there is a need to develop activities that older people can perform conveniently; suspension training and more traditional modes such as resistive bands and bodyweight training are strategies that meet this requirement [8,18]. Previous studies indicate that suspension [8,19,20,21], elastic bands [22,23,24,25], and bodyweight training [26,27] are effective in improving body composition and strength in older people. In particular, due to its effects (core muscle activation, strength, and balance improvements), suspension training may be particularly recommendable for older adults [8], while an alternative to the traditional resistance training performed with dumbbells or weight machines is the use of elastic tubes [28]. However, to the best of our knowledge, only one study has investigated changes in body composition and strength in healthy older people when combining bodyweight and elastic bands training [29]. Additionally, no studies have compared the effectiveness of suspension and traditional resistive training strategies in counteracting the aging effects on body composition, BIVA patterns, and strength in an older population. Therefore, the aim of the present investigation was to compare the effects of 12 weeks of suspension versus combined bodyweight and elastic bands training on the aforementioned parameters in older men.

## 2. Methods

### 2.1. Sample Size and Study Design

The present investigation was designed as a 12-week, three-group, randomized control trial with data collected pre- and post-study. An a priori power analysis was conducted to determine the sample size using statistical software (G*Power v. 3.1.9.2, Stuttgart, Germany). Phase angle and handgrip strength were selected as primary outcomes, and we calculated the effect size from previous studies [7,8,9]. A two-way repeated-measures analysis of variance (ANOVA) was selected as the F test using bioelectrical vector and phase angle, and handgrip strength as main variables with the following criteria: α = 0.05; (1 − β) = 0.8; effect size f = 0.30; correlation among repeated measures = 0.6. Calculation via G*Power determined that a sample size of 27 participants was needed to achieve adequate statistical power. To meet this estimate, we projected an attrition rate of approximately 5 people and thus recruited 35 subjects to participate. After acceptance, the participants were only included in the study after being evaluated by a medical doctor and released without restriction for participation in physical exercise programs. Once approved, participants were randomly allocated to 1 of 3 conditions: (1) a group that performed a suspension training program; (2) a group that performed a training program consisting of bodyweight exercises and elastic tubes, or; (3) a non-exercise control group. Random allocation (random.org) into groups was carried out by a blinded researcher. The exercise programs were performed in an outdoor sports center. The present study followed the CONSORT statement recommendations for reporting randomized trials [30]. The study was approved by the local Ethics Committee of the University of Bologna (approval number: 5315) and was conducted in accordance with the Declaration of Helsinki on research involving human beings. The study was registered as a clinical trial (ClinicalTrials.gov NCT04834804).

### 2.2. Participants

Thirty-six sedentary older men (age 67.4 ± 5.1 years, body weight 76.6 ± 10.7 kg, height 1.68 ± 0.72 m, body mass index 27.1 ± 3.3 kg/m^2^) voluntarily participated in the study. Recruitment occurred through advertisements posted in physiotherapy and sports centers in the area. Prospective participants had to meet the following criteria to qualify for inclusion: (1) not have a chronic disabling disease; (2) not be bedridden, institutionalized or hospitalized; (3) be independently mobile without requiring human assistance, even if requiring the aid of devices such as crutches, walkers, etc.; (4) be without amputations; (5) be 60 years or older. Moreover, prospective participants were excluded if they had a pacemaker or the presence of chronic, uncontrolled metabolic diseases. All participants signed an informed consent after being made aware about the study proposal and procedures.

### 2.3. Intervention Training Programs

**Figure 1:**
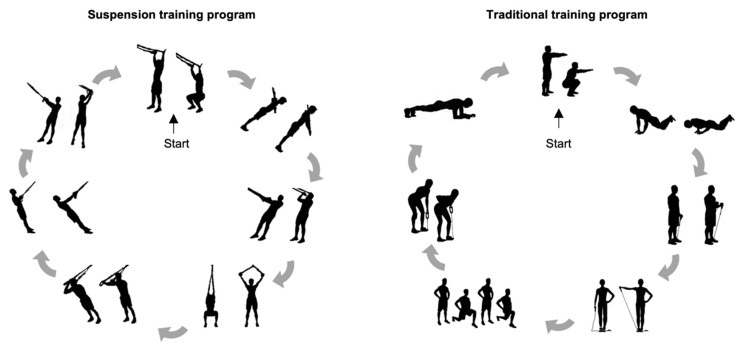
The exercise sequence for the suspension and traditional training programs.

The present investigation lasted a total of 16 weeks, of which 2 weeks (e.g., weeks 2 and 15) were dedicated to assessment and measurements and 12 weeks were allocated to the exercise treatment, for which the Consensus on Exercise Reporting Template (CERT) consensus for the description of the exercise part was followed [31]. The participants underwent two different training programs carried out in thrice-weekly ~60-min sessions on alternate days (Mondays, Wednesdays and Fridays, in the morning). Each session of both training programs consisted of a warm-up, followed by a performance of seven resistive-based exercises, and then concluded with general stretching. The resistance training protocol comprised three sets of twelve repetitions (fifteen seconds for the plank exercise) and a passive rest of 1 min between sets. The Borg rating of perceived exertion (RPE) scale was used to gauge participants’ intensity of effort (from 6 to 20), with the training load targeted at a 13-grade RPE rating as advised by the American College of Sports Medicine [32,33]. When participants perceived the training load at less than 13-grade, we increased the exercise intensity for the next workout session. The exercise order was alternated by body segment to ensure individual muscles had sufficient time to recover between exercises, as shown in Figure 1. The exercises used in the suspension training program were: squat, biceps curl, chest press, low row, rotational ward, squat with Y deltoid fly, and triceps pushdown. The suspension training program was carried out using suspension training straps (TRX, Fitness Anywhere LLC, San Francisco, CA, USA) attached to a fixed beam. The exercises used in the traditional program were: squat, alternating lunge, alternating curl with elastic tube, push up, plank, row with elastic tube, and alternating lateral raise with elastic tube. The elastic bands employed in the traditional training program used different tubes sizes specific to the given exercise (Sidea, Cesena, Italy). The exercise resistance was adjusted by trained coaches who personally supervised all training sessions to ensure safety and adherence to the training protocols. For the suspension training, resistance was increased by altering the body segment inclination related to the ground (i.e., higher inclination equals to higher intensity), whereas we used different sizes of elastic tubes on participants who performed the traditional resistance program. To increase the intensity in bodyweight exercises, participants were required to practice a longer eccentric contraction time.

The participants also were instructed not to participate in any other type of training program during the study period. Participants had to complete at least 75% of the scheduled training sessions for their data to be included in the final analyses.

Food intake was assessed by the 24 h dietary recall method applied on two non-consecutive days of the week [7]. Dietary intake was monitored in the first and last two weeks of the intervention period. A trained nutritionist performed face-to-face interviews with each of the participants and collected detailed descriptions of the type and amount of food consumed on the previous day and on food items that could be easily forgotten. The dietary information was collected in a written format by the nutritionist.

### 2.4. Primary Outcomes: Phase Angle and Handgrip Strength Assessment

Resistance (R) and reactance (Xc) parameters were divided by standing body height in meters. Phase angle was calculated as the arctangent of Xc/R*180°/π. Dominant handgrip strength was measured with a dynamometer (Takei Scientific Instruments Co., Niigata City, Japan) in a sitting position at a 90 degree flexion of their elbow. Each participant performed three trials with a 1 min rest period between each test. The highest value of all three measurements was used for analysis. In order to avoid any confounding effect of time of day [34], all test sessions were performed in the morning.

### 2.5. Secondary Outcomes: Body Composition and Bioimpedance Vector Patterns

Each participant’s height (H) was recorded to the nearest 0.1 cm with a standing stadiometer (GPM, Steckborn, Swiss) and body mass was measured to the nearest 0.1 kg with a high-precision mechanical scale (GPM, Steckborn, Swiss). Body mass index (BMI) was calculated as the ratio of body weight to height squared (kg/m^2^). With the arm relaxed, waist and calf circumferences were taken to the nearest 0.1 cm using a non-stretchable tape measure (GPM, Steckborn, Swiss). All anthropometric data were collected by a physician (F.C.) specifically trained according to a standardized protocol [35]. For each anthropometrical point considered, three non-consecutive measurements were performed in order to extract the average. The technical error of measurement score (TEM) was required to be within 1% for circumferences [36].

The impedance measurements were performed with a bioimpedance analyzer (BIA 101 Anniversary, Akern, Florence, Italy) at a frequency of 50 kHz. The accuracy of the BIA instrument was validated before each test session following the manufacturer’s instructions. The participants were assessed in the supine position with legs (45° compared to the median line of the body) and arms (30° from the trunk) abducted. After cleansing the skin with alcohol, two electrodes were placed on the right hand and two on the right foot. Bioimpedance values were analyzed according to classic and specific BIVA methods [11,13,37,38]. As proposed by Piccoli et al. [11], bioelectrical variables can be analyzed in relation to the distribution of the reference population (through tolerance ellipses). Mean vectors can be compared among groups by means of 95% confidence ellipses. Mean vectors can also correspond to differences between paired observations (in this case, post minus pre training values) and projected as confidence ellipses on the paired R-Xc graph sheet. Body composition parameters were estimated using specific bioimpedance-derived equations [39,40,41] as follows:

Fat-free mass (FFM) (kg) = 1.20 + 0.45 × H^2^/R + 0.18 × body mass

Fat mass (kg) = Body mass − FFM

Total body water (TBW) (kg) =3.75 + 0.45 × H^2^/R+0.11 × body weight

Appendicular skeletal muscle mass (ASMM) (kg) = −3.964 + (0.227 × H^2^/R) + (0.095 × weight) + (1.384 × 1) + (0.064 × Xc)

Skeletal muscle mass index was calculated as the ratio of ASMM to height squared (kg/m^2^).

### 2.6. Statistical Analysis

Data were statistically analyzed with SPSS v. 27.0 (SPSS, IBM Corp., Armonk, NY, USA). The Kolmogorov–Smirnov test was used to check the normal distribution of data. A one-way ANOVA was used to assess whether participants differed in age and BMI at baseline. A two-way repeated-measures ANOVA was performed to determine the changes over time in body composition and dominant handgrip strength, considering specific within (time, pre-post intervention) and between factors (treatment, three groups). For analysis of variance outcomes, effect size (ES) was assessed by using partial eta squared (η_p_^2^). In cases where the F-value was significant (*p* < 0.05), multiple comparisons were performed to examine changes across the 12 weeks using the Bonferroni correction. The paired, one-sample Hotelling’s T^2^ test, a multivariate extension of the Student’s t test for paired data, was performed to determine if the changes in the mean group vectors were significantly different from zero (null vector). Hedges’ d effect size was calculated for the pairwise comparisons. Mahalanobis distance (D^2^), which represents a multivariate measure of effect and a multivariate measure of distance, was calculated to determine the magnitude of the changes in the mean group vectors. Intraclass correlation coefficients, (ICC) and standard error of measurement (SEM) were calculated for the primary outcomes. Significance was set with *p* ≤ 0.05.

## 3. Results

**Figure 2:**
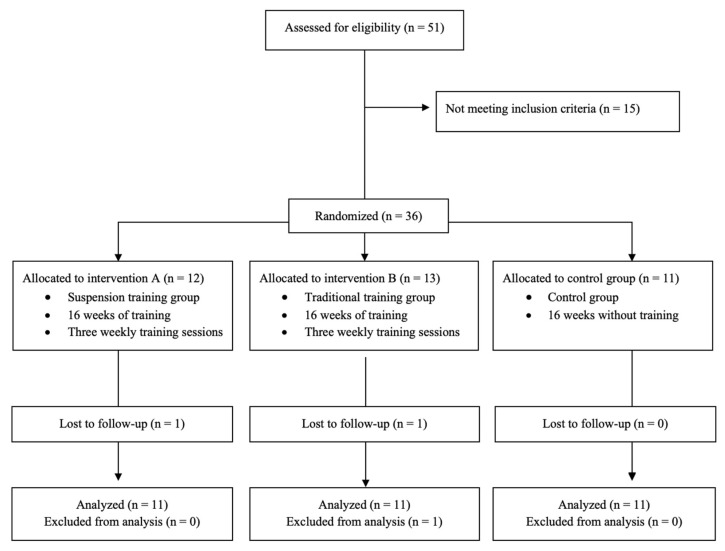
Flow chart.

The flow chart with a schematic representation of the participant allocation is shown in Figure 2. Two participants abandoned the training programs for personal reasons, while one participant did not complete the required number of traditional training sessions. No significant (*p* > 0.05) differences in age (F = 0.775, *p* = 0.469) and BMI (*t* = 0.177, *p* = 0.839) were found between the three groups at baseline. Comparisons of body composition, bioelectrical, and strength parameters between the groups at baseline were reported in Table S1.

**Figure 3:**
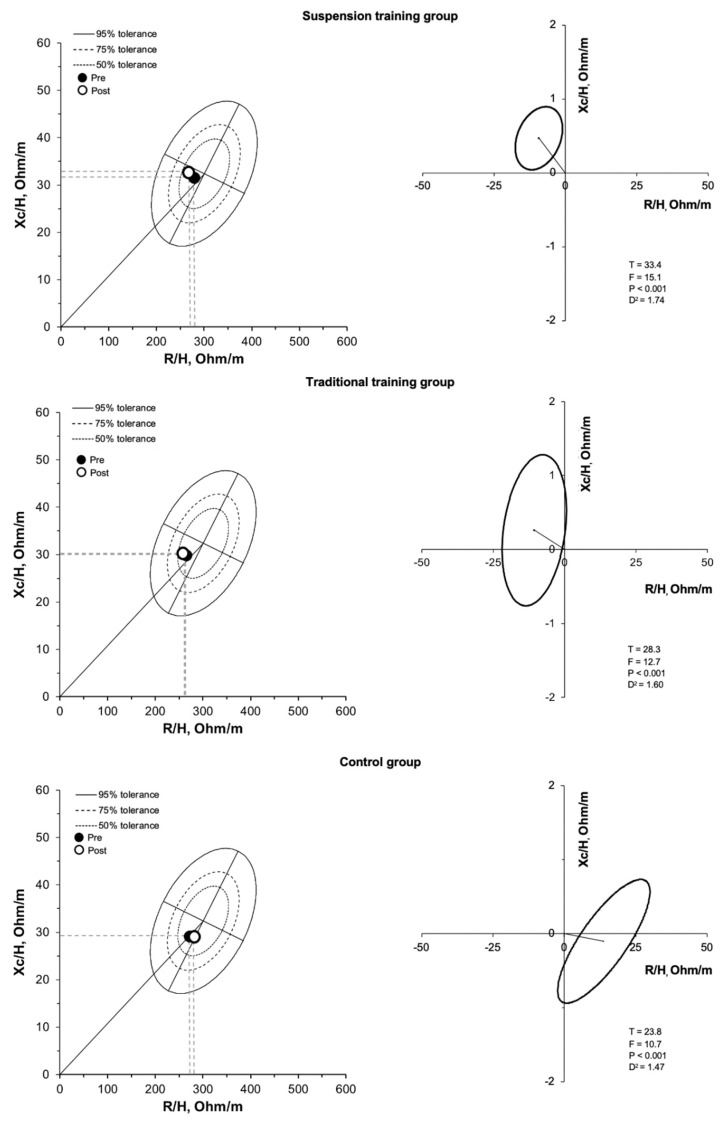
R-Xc and paired graphs for the multivariate changes in classic resistance and reactance are shown. On the left panels, bioimpedance data are plotted on the tolerance ellipses of the reference population. On the right panels, mean vector displacements with 95% confidence ellipses and results of the Hotelling’s T^2^ test are shown.

**Figure 4:**
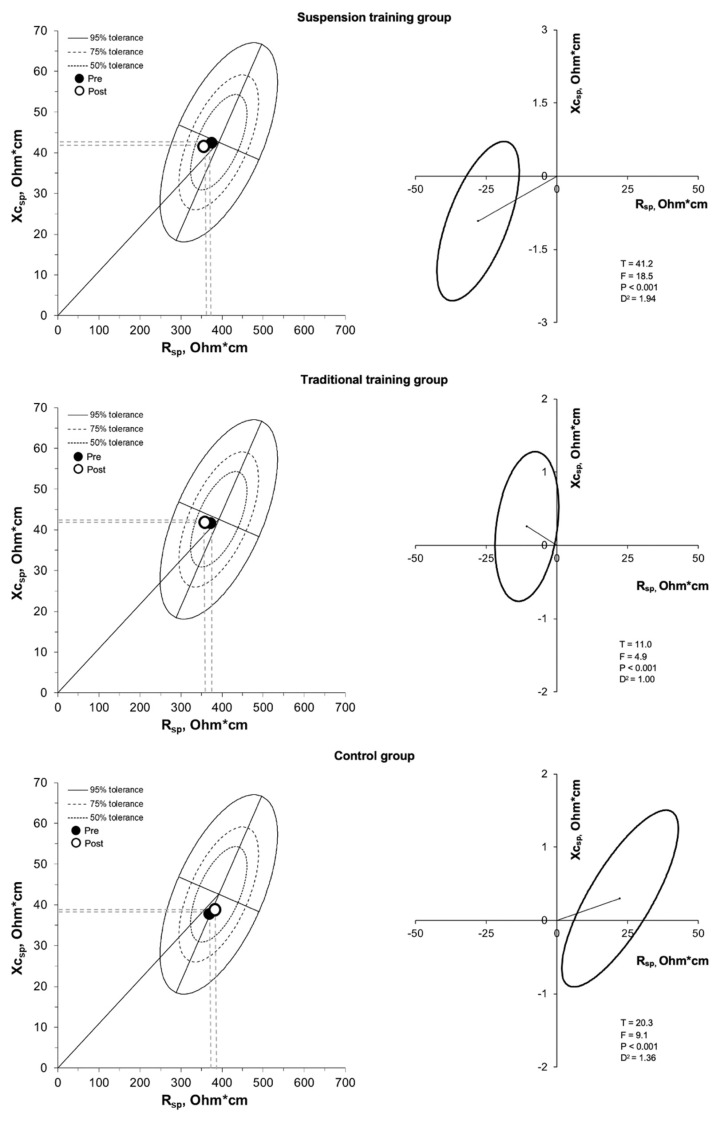
R-Xc and paired graphs for the multivariate changes in specific resistance and reactance are shown. On the left panels, bioimpedance data are plotted on the tolerance ellipses of the reference population. On the right panels, mean vector displacements with 95% confidence ellipses and results of the Hotelling’s T^2^ test are shown.

Considering the primary outcomes, the ICC was 0.819 and 0.991 for phase angle and dominant handgrip strength, respectively; SEM was 0.012 degrees and 0.092 kg for phase angle and dominant handgrip strength, respectively. There was a significant (*p* < 0.05) group by time interaction for absolute and relative fat mass, fat free mass, total body water, appendicular skeletal muscle mass, skeletal muscle mass index, handgrip strength, and bioelectrical classic and specific resistance, classic reactance, phase angle, even after adjusting for age, BMI, and baseline values (Table 1). Absolute and relative fat mass decreased in the participants who performed the suspension training program, did not show a statistically significant difference in the traditional training group, and showed an increase in the control group (Table 1). The BIVA analysis, using both the classic (Figure 3, Table 1) and specific approach (Figure 4, Table 1), showed significant differences when comparing body composition pre- and post-training in all the groups. In the suspension group, classic and specific BIVA showed a significant reduction in vector length after training, indicative of an increase in total body water and a reduction in percentage of fat mass, respectively, and an increase in phase angle, indicative of an increase in intracellular/extracellular water ratio and skeletal muscle mass. The traditional training group showed an increase in phase angle only (higher skeletal muscle mass; higher intracellular/extracellular water ratio), while in the control group an opposite effect was observed, with an increase in specific vector length (greater percentage of fat mass) and a reduction in phase angle (lower skeletal muscle mass; lower intracellular/extracellular water ratio).

**Figure 5:**
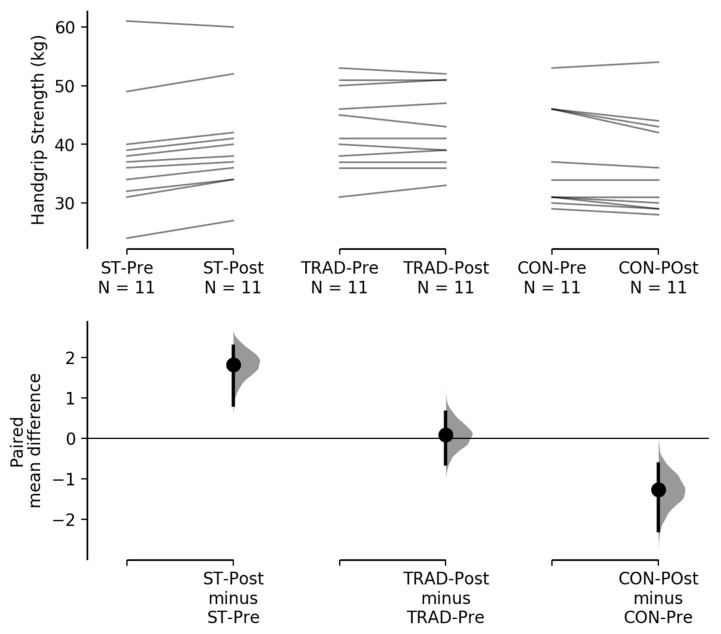
The paired mean difference for 3 comparisons are shown in the above Cumming estimation plot [42]. The raw data are plotted on the upper axes; each paired set of observations is connected by a line. On the lower axes, each paired mean difference is plotted as a bootstrap sampling distribution. Mean differences are depicted as dots; 95% confidence intervals are indicated by the ends of the vertical error bars.

Handgrip strength increased and decreased in the suspension training and control groups, respectively, and remained stable in the traditional training group (Table 1 and Figure 5).

## 4. Discussion

The main purpose of this investigation was to compare the longitudinal effects of suspension and traditional training on body composition, BIVA patterns, and handgrip strength in older men. Although suspension, bodyweight and elastic band training counteracted the aging-related decline in the investigated parameters, only the suspension training program improved markers of body composition and handgrip strength after the 12-week intervention.

The suspension training protocol induced beneficial changes in fat mass, total body water, appendicular skeletal muscle mass, and skeletal muscle index. Consistent with our outcomes, a previous study [8] found similar results regarding a reduction in fat mass in older people after 12 weeks of suspension training. However, to the best of our knowledge, no previous study has investigated the effects of suspension training on other body composition parameters such as total body water and skeletal muscle index. As opposed to the suspension training protocol, training with resistive bands and bodyweight was not effective in improving the investigated body composition parameters. In contrast to our findings, some studies have reported decreases in body fat percentage and increases in appendicular skeletal muscle mass after 12 weeks of elastic band training [22]. Discrepancies between findings potentially may be explained by differences in the respective sample populations; we studied healthy male adult participants whereas they assessed adult female patients identified as having sarcopenic obesity. Therefore, their lower baseline muscle mass and higher fat mass may have resulted in different outcomes.

To the best of our knowledge, no studies have investigated the effects of a bodyweight training protocol on body composition in an older population, and no studies using bodyweight and/or elastic bands have investigated parameters such as total body water and skeletal muscle index. Our results suggest that suspension training could be more effective in improving body composition than the combination of bodyweight and resistive band exercise training. These findings may be attributable to the fact that suspension training involves factors that require a high energy expenditure, such as proprioceptive stimuli, constant core activation, and arm isometric contraction [21]. The classic BIVA showed changes in total body water, and specific BIVA changes in fat mass percentage, while both approaches gave information on muscle mass changes. The reduction of phase angle (proxy of muscle mass) observed in the control group is in agreement with the age-related decrease previously observed using both the classic [12] and specific approach [36], and highlights the relevance of its increase in the exercise groups.

We observed an increase in handgrip strength after the 12 weeks of suspension training. Several studies showed similar outcomes on dominant handgrip strength in older people using different suspension training protocols, such as high-intensity interval training [8,19]. Alternatively, the traditional training program did not increase dominant handgrip strength in participants. Consistent with this finding, other studies show no change in handgrip strength after 12 weeks of traditional training [29,43]. The reasons for discrepancies between modalities on this outcome are not clear, but may be due to the specific composition of variables in the respective programs; further investigation is warranted on the topic. However, our results show that the traditional training program was able to attenuate the decline in handgrip strength, as observed in the control group. Furthermore, as the exercise programs suggested in this study, other home-based training strategies, such as Wii^®^ games, have been identified as improving health status in the elderly [16,17].

This investigation presents some limitations that should be taken into account. First, our results are specific to elderly men and thus not generalizable to other populations such as elderly women, younger individuals, and those with clinical conditions. Second, although participants were instructed to maintain their usual lifestyle habits, we were unable to monitor physical activity levels outside of the study environment; thus, it is possible that differences in this variable may have confounded results. Third, a possible confounding variable would be that the groups did not train at the same intensity and therefore the results would have been different when volumes and intensities were equal. Lastly, our results cannot be compared with those obtained from bioimpedance measurements performed using different technology and sampling frequency than the ones used here.

### Practical Applications

Suspension training may be a potent stimulus influencing body composition, bioimpedance vector patterns, and handgrip strength in older adults. Suspension exercise training programs induce handgrip strength increase, and this may be a consequence of the constant grip on the handles required when using the suspension trainer tool. Resistance body weight and elastic tube training offers a potential means for preserving body composition and improving handgrip strength in old age. Although resistance training using bodyweight exercises and elastic tubes helps to prevent a decline in body composition, suspension training appears to be more effective in elderly men. Elderly subjects could potentially benefit from these training strategies as they involve the use of low-cost tools and can be performed at home instead of in specialized sports centers. Lastly, specific and classic BIVA may identify body composition changes over time, avoiding the use of expensive methods or procedures. In addition, the evaluation of phase angle for assessing the effect of training strategies on cellular integrity and nutritional status represents an interesting topic for future research on sports nutrition.

## 5. Conclusions

It is known that aging represents an irreversible condition for body composition and strength; therefore, elderly people should be encouraged to take part in systematic resistance training. The use of suspension tools in a home-based setting might help to attenuate negative effects of aging by improving measures of body composition and strength, and thus provide a convenient alternative to more traditional resistance practices.

## Figures and Tables

**Table 1:** The baseline (mean ± standard deviation) and post 12 weeks values (mean ± standard deviation) of the dependent parameters are shown.

Variable		Suspension Training Group (*n* = 11)	Traditional Training Group (*n* = 11)	Control Group (*n* = 11)	Group x Time Interaction
Phase angle (degree)	Baseline	6.5 ± 0.6	6.5 ± 0.7	6.1 ± 0.6	F = 24.4, *p* < 0.001, η_p_^2^ = 0.62
	After 12 weeks	6.8 ± 0.7 *	6.8 ± 0.8 *	5.8 ± 0.4 *	
Dominant handgrip strength (kg)	Baseline	38.2 ± 9.7	42.3 ± 8.4	37.6 ± 8.5	F = 16.9, *p* < 0.001, η_p_^2^ = 0.53
	After 12 weeks	40.1 ± 9.0 *	42.6 ± 8.5	36.4 ± 8.4 *	
Fat mass (kg)	Baseline	16.7 ± 5.1	18.9 ± 6.3	16.1 ± 2.9	F = 14.5, *p* < 0.001, η_p_^2^ = 0.49
	After 12 weeks	15.4 ± 5.1 *	18.0 ± 5.4	17.9 ± 2.9 *	
Fat mass (%)	Baseline	23.2 ± 5.9	22.6 ± 5.1	21.9 ± 3.2	F = 15.1, *p* < 0.001, η_p_^2^ = 0.50
	After 12 weeks	21.4 ± 6.1 *	21.7 ± 4.5	24.2 ± 3.1 *	
Fat-free mass (kg)	Baseline	54.6 ± 4.1	63.1 ± 8.9	57.3 ± 4.2	F = 9.2, *p* = 0.001, η_p_^2^ = 0.38
	After 12 weeks	55.4 ± 3.9	63.9 ± 3.9	55.9 ± 3.5	
Total body water (kg)	Baseline	39.9 ± 3.1	46.0 ± 6.9	41.9 ± 3.1	F = 9.8, *p* = 0.001, η_p_^2^ = 0.39
	After 12 weeks	40.7 ± 3.0 *	46.8 ± 7.2	40.7 ± 2.4	
Appendicular skeletal muscle mass (kg)	Baseline	20.7 ± 1.4	23.6 ± 3.3	21.4 ± 1.5	F = 12.4, *p* < 0.001, η_p_^2^ = 0.45
	After 12 weeks	21.1 ± 1.3 *	24.0 ± 3.5	20.7 ± 1.3	
Skeletal muscle index (kg/m^2^)	Baseline	7.7 ± 0.5	8.0 ± 1.2	7.6 ± 0.3	F = 11.9, *p* < 0.001, η_p_^2^ = 0.44
	After 12 weeks	7.9 ± 0.5 *	8.2 ± 1.2	7.4 ± 0.2	
R/H (ohm/m)	Baseline	285.9 ± 22.9	263.9 ± 35.5	274.8 ± 16.3	F = 11.4, *p* < 0.001, η_p_^2^ = 0.43
	After 12 weeks	276.8 ± 21.6 *	258.0 ± 35.8	286.9 ± 10.5	
Xc/H (ohm/m)	Baseline	32.3 ± 5.0	29.9 ± 3.9	29.4 ± 2.3	F = 3.40, *p* < 0.046, η_p_^2^ = 0.18
	After 12 weeks	33.1 ± 5.1 *	30.3 ± 4.0	29.4 ± 2.1	
R_sp_ (ohm*cm)	Baseline	384.9 ± 38.8	370.3 ± 51.5	364.4 ± 49.4	F = 24.1, *p* < 0.001, η_p_^2^ = 0.61
	After 12 weeks	357.1 ± 33.4 *	359.7 ± 44.7	386.7 ± 38.2 *	
Xc_sp_ (ohm*cm)	Baseline	43.5 ± 7.2	42.3 ± 8.4	38.8 ± 5.7	F = 2.67, *p* = 0.086, η_p_^2^ = 0.15
	After 12 weeks	42.6 ± 6.5	42.6 ±8.5	39.1 ± 4.7	

Note: *: significantly (*p* < 0.05) different from baseline. R/H: resistance divided by body height, Xc/H: reactance divided by body height, R_sp_: resistance divided by body geometries, Xc_sp_: reactance divided by body geometries, η_p_^2^: partial eta squared.

## Data Availability

The data that support the findings of this study are available from the corresponding author, upon reasonable request.

## Supplementary Materials

The following are available online at https://www.mdpi.com/article/10.3390/nu13072267/s1, Table S1: Comparisons of body composition, bioelectrical and strength parameters between the three groups at baseline.
