# Supplementary material for: Effects of a 12-Week Suspension versus Traditional Resistance Training Program on Body Composition, Bioimpedance Vector Patterns, and Handgrip Strength in Older Men: A Randomized Controlled Trial
Source: Nutrients. 2021 Jun 30;13(7):2267. doi: 10.3390/nu13072267 (PMC8308438; doi:10.3390/nu13072267)
Supplement: Supplementary file 1 [file nutrients-13-02267-s001.zip › nutrients-1273873-supplementary.pdf]

Table S1. Comparisons of body composition, bioelectrical and strength parameters between the three groups at baseline.

Variable		Suspension training group (n=11)	Traditional training group (n=11)	Control group (n=11)	One-way ANOVA
Fat mass (kg)	Baseline	16.7 ± 5.1	18.9 ± 6.3	16.1 ± 2.9	F: 1.3, P: 0.274
Fat mass (%)	Baseline	23.2 ± 5.9	22.6 ± 5.1	21.9 ± 3.2	F: 0.3, P: 0.755
Fat-free mass (kg)	Baseline	54.6 ± 4.1	63.1 ± 8.9	57.3 ± 4.2	F: 3.6, P: 0.038
Total body water (kg)	Baseline	39.9 ± 3.1	46.0 ± 6.9	41.9 ± 3.1	F: 3.2, P: 0.055
Appendicular skeletal muscle mass (kg)	Baseline	20.7 ± 1.4	23.6 ± 3.3	21.4 ± 1.5	F: 3.5, P: 0.041
Skeletal muscle index (kg/m^2)	Baseline	7.7 ± 0.5	8.0 ± 1.2	7.6 ± 0.3	F: 0.7, P: 0.513
R/H (ohm/m)	Baseline	285.9 ± 22.9	263.9 ± 35.5	274.8 ± 16.3	F: 1.1, P: 0.332
Xc/H (ohm/m)	Baseline	32.3 ± 5.0	29.9 ± 3.9	29.4 ± 2.3	F: 1.4, P: 0.264
R_{sp} (ohm*cm)	Baseline	384.9 ± 38.8	370.3 ± 51.5	364.4 ± 49.4	F: 0.6, P: 0.595
Xc_{sp} (ohm*cm)	Baseline	43.5 ± 7.2	42.3 ± 8.4	38.8 ± 5.7	F: 1.3, P: 0.274
Phase angle (degree)	Baseline	6.5 ± 0.6	6.5 ± 0.7	6.1 ± 0.6	F: 1.1, P: 0.329
Dominant handgrip strength (kg)	Baseline	38.2 ± 9.7	42.3 ± 8.4	37.6 ± 8.5	F: 1.7, P: 0.205
